# Draft Genome Sequences of Six Isolates of the Bacillus cereus Group Isolated from Pet Reptiles

**DOI:** 10.1128/mra.00385-22

**Published:** 2022-08-15

**Authors:** Milena Skóra, Magdalena Zając, Renata Kwit, Magdalena Skarżyńska, Paulina Pasim, Emilia Mikos-Wojewoda, Arkadiusz Bomba, Aleksandra Giza, Olivier Chesneau, Rene S. Hendriksen, Dariusz Wasyl

**Affiliations:** a Department of Microbiology, National Veterinary Research Institute, Pulawy, Poland; b Department of Omic Analyses, National Veterinary Research Institute, Pulawy, Poland; c Collection de l’Institut Pasteur (CIP), Department of Microbiology, Institut Pasteur, Paris, France; d Research Group for Genomic Epidemiology, WHO Collaborating Centre for Antimicrobial Resistance in Foodborne Pathogens, Food and Agriculture Organization Reference Laboratory for Antimicrobial Resistance, European Union Reference Laboratory for Antimicrobial Resistance, National Food Institute, Technical University of Denmark, Kgs. Lyngby, Denmark; University of Rochester School of Medicine and Dentistry

## Abstract

Bacteria of the Bacillus cereus group are Gram-positive rods and are widespread in nature, but little information is currently available about their presence in reptiles. Here, we report draft genome sequences of six *Bacillus* isolates belonging to three species, namely, Bacillus cereus, Bacillus paranthracis, and Bacillus toyonensis, isolated from pet reptiles in Poland.

## ANNOUNCEMENT

The Bacillus cereus group consists of Gram-positive, rod-shaped, aerobic, or facultatively anaerobic bacteria that belong to 21 closely related species (1, 2). One of the most relevant characteristics of the *Bacillus* genus is the ability of the bacteria to form spores which allow them to survive under harsh environmental conditions. The bacilli are widely distributed in the environment, being present in the soil, water, and other ecological niches (3). The presence of *Bacillus* species was also confirmed in the feces of cows as well as in the gastrointestinal tracts of poultry (4). In this study, we present draft genome sequences of six fecal isolates belonging to three species, namely, Bacillus cereus, Bacillus paranthracis, and Bacillus toyonensis, and compare these sequences with references genomes deposited in GenBank (5).

Fecal samples were incubated in buffered peptone water (BWP) for 18 h at 37°C, and then 10 μL of each culture was streaked onto polymyxin pyruvate-egg yolk-mannitol-bromothymol blue agar (PEMBA) (homemade) and incubated for 24 h at 37°C (6). Single colonies were passaged onto the nutrition agar and incubated overnight at 37°C (7). The pure culture was identified by matrix-assisted laser desorption ionization–time of flight mass spectrometry (MALDI-TOF MS) using the extraction method following the manufacturers’ guidelines (Bruker Daltonik GmbH). After the incubation onto the nutrition agar at 37°C for 24 h, genomic DNA was extracted with the Maxwell Rapid Sample Concentrator (RSC) cultured cells DNA kit (Promega) according to the manufacturer’s instructions. The concentration and quality parameters of the DNA were defined with a spectrophotometer (NanoDrop One) and a Qubit 3 fluorometer. The DNA libraries were prepared using the Nextera XT sample preparation kit following the manufacturer’s recommendations. Sequencing was performed using the MiSeq reagent kit on the MiSeq platform (Illumina) with the 2 × 300-bp paired-end protocol, to 100× depth of sequencing. All bioinformatic tools were used with default parameters, except where otherwise noted. Raw paired-end reads were quality filtered using FastQC v0.11.5 (http://www.bioinformatics.babraham.ac.uk/projects/fastqc) (8), and trimming and adapter sequence removal were executed using Trimmomatic 0.36 (ILLUMINACLIP: 2:30:10, LEADING:3, TRAILING:3, SLIDINGWINDOW:4:15, MINLEN:36) (9). Trimmed reads were then merged using BBMerge from the bbtools software suite (https://jgi.doe.gov/data-and-tools/software-tools/bbtools/bb-tools-user-guide/bbmerge-guide/) and assembled using SPAdes v3.9.0 with the “-careful” flag (10). The draft genome sizes varied from 5,135,335 to 5,937,563 bp, and the GC content was approximately 35.1%. Genomic features (number of contigs, *N*_50_ value, GC content, and total size) were defined with QUAST (11) and included in Table 1. Average nucleotide identity (ANI) was analyzed using the JSpeciesWS online service (12) which revealed that the PIW23 genome sequence had the highest ANI based on MUMer (ANIm) value to *B. paranthracis* (GenBank accession number GCF_001883995.1; 99.23%); PIW25 and PIW27 to *B. toyonensis* (GCA_000496285.1; >99.58%); and PIW26, PIW28, and PIW162 to B. cereus (GCA_000007825.1; >96.05%). The multilocus sequence type (MLST) was validated by using MLST 2.0 (https://cge.cbs.dtu.dk/services/MLST/) (13). The MLST scheme for B. cereus was applied to all isolates. The MLST type was found in PIW25, PIW27, PIW28, and PIW162, and other isolates (PIW23, PIW26) were identified as an unknown sequence type (ST).

**TABLE 1:** Genome properties of the *Bacillus* strains isolated from reptiles in Poland

Isolate	Species	Host	Genome size (bp)	No. of contigs	Total no. of reads	Coverage	GC content (%)	*N*_50_ (bp)	MLST type	SRA accession no.	Genome accession no.
PIW23	Bacillus paranthracis	Eumeces schneideri	5,135,275	35	3,949,460	205×	35.3	1,174,839	Unknown (nearest STs: 869, 182, 761)	SRR18728991	JAKJQA000000000
PIW25	Bacillus toyonensis	Testudo hermanni	5,937,563	58	4,533,936	208×	35.0	648,267	ST972	SRR18728986	JAKJPV000000000
PIW26	Bacillus cereus	Testudo hermanni	5,866,545	103	2,083,574	89×	34.9	334,014	Unknown (nearest STs: 1197, 193)	SRR18728987	JAKJPW000000000
PIW27	Bacillus toyonensis	Testudo horsfieldii	5,646,249	63	3,602,000	192×	35.0	556,185	ST718	SRR18728988	JAKJPX000000000
PIW28	Bacillus cereus	Testudo horsfieldii	5,534,170	93	3,694,768	200×	35.0	328,652	ST197	SRR18728989	JAKJPY000000000
PIW162	Bacillus cereus	Testudo horsfieldii	5,442,449	29	4,761,076	263×	35.1	1,736,072	ST511	SRR18728990	JAKJPZ000000000

### Data availability.

The draft genome sequences reported here were deposited in GenBank under the BioProject accession number PRJNA799608. The raw sequence read and genome assembly accession numbers are listed in Table 1.

## References

[1] Liu Y, Du J, Lai Q, Zeng R, Ye D, Xu J, Shao Z. 2017. Proposal of nine novel species of the Bacillus cereus group. Int J Syst Evol Microbiol 67:2499–2508. doi:10.1099/ijsem.0.001821. 28792367

[2] Bukharin OV, Perunova NB, Andryuschenko SV, Ivanova EV, Bondarenko TA, Chainikova IN. 2019. Genome sequence announcement of Bacillus paranthracis strain ICIS-279, isolated from human intestine Microbiol Resour Announc 8:e00662-19. doi:10.1128/MRA.00662-19. 31672737 PMC6953507

[3] Fira D, Dimkić I, Berić T, Lozo J, Stanković S. 2018. Biological control of plant pathogens by Bacillus species. J Biotechnol 285:44–55. doi:10.1016/j.jbiotec.2018.07.044. 30172784

[4] Nfor NN, Lapin CN, Richard Mclaughlin W. 2015. Isolation of Bacillus cereus group from the fecal material of endangered wood turtles. Curr Microbiol 71:524–527. doi:10.1007/s00284-015-0875-x. 26175111

[5] Karen Clark G, Karsch-Mizrachi I, Lipman DJ, Ostell J, Sayers EW. 2016. Genbank. Nucleic Acids Res 44:D67–D72. doi:10.1093/nar/gkv1276. 26590407 PMC4702903

[6] International Organization for Standardization. 2006. ISO 21871:2006. Microbiology of food and feed stuffs. Horizontal method for the determination of low numbers of presumptive Bacillus cereus—most probable number technique and detection method. International Organization for Standardization, Geneva, Switzerland.

[7] International Organization for Standardization. 2014. ISO/TR 6579–3:2014(E): microbiology of the food chain: horizontal method for the detection, enumeration and serotyping of Salmonella. Part 3. Guidelines for serotyping of Salmonella spp. International Organization for Standardization, Geneva, Switzerland.

[8] Andrews S. 2010. FastQC: a quality control tool for high throughput sequence data. https://www.bioinformatics.babraham.ac.uk/projects/fastqc/.

[9] Bolger AM, Lohse M, Usadel B. 2014. Genome analysis Trimmomatic: a flexible trimmer for Illumina sequence data. Bioinformatics 30:2114–2120. doi:10.1093/bioinformatics/btu170. 24695404 PMC4103590

[10] Bankevich A, Nurk S, Antipov D, Gurevich AA, Dvorkin M, Kulikov AS, Lesin VM, Nikolenko SI, Pham S, Prjibelski AD, Pyshkin AV, Sirotkin AV, Vyahhi N, Tesler G, Alekseyev MA, Pevzner PA. 2012. SPAdes: a new genome assembly algorithm and its applications to single-cell sequencing. J Comput Biol 19:455–477. doi:10.1089/cmb.2012.0021. 22506599 PMC3342519

[11] Gurevich A, Saveliev V, Vyahhi N, Tesler G. 2013. Genome analysis QUAST: quality assessment tool for genome assemblies. Bioinformatics 29:1072–1075. doi:10.1093/bioinformatics/btt086. 23422339 PMC3624806

[12] Richter M, Rossell O-M, Ora R, Glö Ckner FO, Rg Peplies J. 2016. JSpeciesWS: a Web server for prokaryotic species circumscription based on pairwise genome comparison. Bioinformatics 32:929–931. doi:10.1093/bioinformatics/btv681. 26576653 PMC5939971

[13] Larsen MV, Cosentino S, Rasmussen S, Friis C, Hasman H, Marvig RL, Jelsbak L, Sicheritz-Pontén T, Ussery DW, Aarestrup FM, Lund O. 2012. Multilocus sequence typing of total-genome-sequenced bacteria. J Clin Microbiol 50:1355–1361. doi:10.1128/JCM.06094-11. 22238442 PMC3318499

